# Intrapulmonary administration of recombinant activated factor VII in pediatric, adolescent, and young adult oncology and hematopoietic cell transplant patients with pulmonary hemorrhage

**DOI:** 10.3389/fonc.2024.1375697

**Published:** 2024-04-12

**Authors:** Caitlin Hurley, Jennifer McArthur, Jeffrey M. Gossett, Elizabeth A. Hall, Patricia J. Barker, Diego R. Hijano, Melissa R. Hines, Guolian Kang, Jason Rains, Saumini Srinivasan, Ali Suliman, Amr Qudeimat, Saad Ghafoor

**Affiliations:** ^1^ Department of Pediatrics, Division of Critical Care Medicine, St. Jude Children’s Research Hospital, Memphis, TN, United States; ^2^ Department of Bone Marrow Transplant and Cellular Therapy, St. Jude Children’s Research Hospital, Memphis, TN, United States; ^3^ Department of Biostatistics, St. Jude Children’s Research Hospital, Memphis, TN, United States; ^4^ Department of Clinical Pharmacy and Translational Science, University of Tennessee Health Science Center, Memphis, TN, United States; ^5^ Department of Pharmacy and Pharmaceutical Sciences, St. Jude Children’s Research Hospital, Memphis, TN, United States; ^6^ Department of Infectious Diseases, St. Jude Children’s Research Hospital, Memphis, TN, United States; ^7^ Department of Pediatrics, University of Tennessee Health and Science Center, Memphis, TN, United States; ^8^ Department of Pediatrics, Division of Pulmonary Medicine, University of Tennessee Health and Science Center, Memphis, TN, United States

**Keywords:** pulmonary hemorrhage, diffuse alveolar hemorrhage (DAH), hematopoietic cell transplant (HCT), recombinant factor VIIa, pediatric oncologic emergencies, critical care

## Abstract

**Introduction:**

Diffuse alveolar hemorrhage (DAH) is a devastating disease process with 50-100% mortality in oncology and hematopoietic cell transplant (HCT) recipients. High concentrations of tissue factors have been demonstrated in the alveolar wall in acute respiratory distress syndrome and DAH, along with elevated levels of tissue factor pathway inhibitors. Activated recombinant factor VII (rFVIIa) activates the tissue factor pathway, successfully overcoming the tissue factor pathway inhibitor (TFPI) inhibition of activation of Factor X. Intrapulmonary administration (IP) of rFVIIa in DAH is described in small case series with successful hemostasis and minimal complications.

**Methods:**

We completed a single center retrospective descriptive study of treatment with rFVIIa and outcomes in pediatric oncology and HCT patients with pulmonary hemorrhage at a quaternary hematology/oncology hospital between 2011 and 2019. We aimed to assess the safety and survival of patients with pulmonary hemorrhage who received of IP rFVIIa.

**Results:**

We identified 31 patients with pulmonary hemorrhage requiring ICU care. Thirteen patients received intrapulmonary rFVIIa, while eighteen patients did not. Overall, 13 of 31 patients (41.9%) survived ICU discharge. ICU survival (n=6) amongst those in the IP rFVIIa group was 46.2% compared to 38.9% (n=7) in those who did not receive IP therapy (p=0.69). Hospital survival was 46.2% in the IP group and 27.8% in the non-IP group (p=0.45). There were no adverse events noted from use of IP FVIIa.

**Conclusions:**

Intrapulmonary rFVIIa can be safely administered in pediatric oncology patients with pulmonary hemorrhage and should be considered a viable treatment option for these patients.

## Introduction

Pulmonary hemorrhage, specifically, diffuse alveolar hemorrhage (DAH), is a devastating disease process with 50-100% mortality in oncology and hematopoietic cell transplant (HCT) recipients. It is characterized by a pattern of clinical and radiological findings including hypoxemic respiratory failure, anemia, hemoptysis, diffuse interstitial infiltrates on chest radiography, and progressively bloody return on bronchoalveolar lavage (1–4). The pathogenesis, while not fully elucidated, is thought to occur from direct lung injury of varying etiologies, leading to alveolar inflammation, dysregulated cytokine release, and subsequent widespread injury to the alveolar-capillary basement membrane (1, 2, 5). Pulmonary inflammation causes increased intra-alveolar expression of tissue factor (TF) with high concentrations detected in the alveolar wall in patients with acute respiratory distress syndrome (ARDS), pneumonia, and DAH. Tissue factor pathway inhibitors (TFPIs) also increase significantly. TFPIs prevent binding of activated Factor VII (FVIIa) and TF, ultimately preventing Factor X activation and downstream activation of fibrin to achieve hemostasis. This phenomenon offers a hypothesis for increased risk of bleeding in the inflamed lung as well as rationale for local administration of hemostatic agents (6–8). In addition to overcoming the TFPI, Factor VIIa is useful in patients who lack abnormalities on traditional coagulation studies. Factor VIIa not only directly activates Factor X, but it also increases thrombin production on the surface of activated platelets without the need of VIII and IX, and even in the face of in thrombocytopenia (9–13).

As DAH in oncology and HCT patients is thought to be propagated from dysregulated inflammation, glucocorticoids have been the mainstay of therapy, though its use has not resulted in significantly improved outcomes (2, 4, 14–16). Novel administration of hemostatic agents, such as inhaled tranexamic acid (TXA) and activated recombinant factor VII (rFVIIa), in oncology and post-HCT DAH has yielded promising preliminary outcomes in few adult and pediatric case reports and case series (7, 8, 17–23). These potentially promising results prompted the recent incorporation of these agents into our clinical practice for patients at very high risk of death. We present a retrospective review of patients over 8 years during which our treatment practice has evolved. We hypothesized that the use of intrapulmonary rFVIIa would be safe and improve survival.

## Methods

We completed a single-center, retrospective descriptive study of treatment regimens and outcomes in pediatric, adolescent, and young adult oncology and HCT patients diagnosed with pulmonary hemorrhage, including DAH, at a quaternary pediatric hematology/oncology hospital (St. Jude Children’s Research Hospital, Memphis, TN, USA) between August 2011 and December 2019. The study underwent expedited review approval by the local Institutional Review Board. Patients were identified by ICU admission log diagnoses and electronic medical record survey of codes for pulmonary hemorrhage and diffuse alveolar hemorrhage. Treatment regimens were extracted from the medical record including date, time, route of administration, and dose of rFVIIa as well as additional adjuvant therapies with steroids, immunomodulators and inhaled TXA. Dose routes were characterized as intravenous (IV) or intrapulmonary. Intrapulmonary was defined as nebulized, direct instillation via ETT with or without bronchoscopy. Demographic and outcome data were collected including primary diagnosis, history, and type of HCT, ventilator free days, ICU and hospital length of stay, platelet counts and coagulation panels at onset of hemorrhage. We also collected safety data including need for reintubation for endotracheal tube obstruction from thrombosis or secretions. The primary outcome was defined as survival to ICU discharge.

### Drug preparation and administration

Activated human recombinant factor VII (rFVIIa) solutions were aseptically prepared by the inpatient pharmacy and dispensed to the ICU. For direct intrapulmonary administration during bronchoscopy procedures patients received rFVIIa 50mcg/kg/dose (rounded to nearest vial size). As previously described, the dose was diluted with 25-50 ml of sterile 0.9% sodium chloride solution and divided into 5 aliquots of approximately 5-10 ml to facilitate ease of administration into the five lobes of the lung (7, 18). For nebulization, rFVIIa 50-75 mcg/kg/dose (rounded to nearest vial size) was prepared in 3-5 ml of sterile 0.9% sodium chloride and administered at varying frequency from every 6 hours to once daily at the discretion of the prescribing physician. The Aerogen Solo™ vibrating mesh nebulizer was placed inline before the humidifier on the inspiratory side of the ventilator circuit. After encountering problems with ventilator malfunction during inhaled TXA delivery in prior patients, the following procedure was implemented. Despite nebulized delivery of rFVIIa not reported to have the same effects as TXA on the ventilator circuit, to ensure safe delivery, we employed the following precautions with administration of all inhaled agents including rFVIIa. Two Maquet Servo Duo guard filters were placed on the expiratory limb of the ventilator circuit and exchanged immediately upon completion of medication delivery (**Supplementary Figure 1A**). A one-way valve was placed between the nebulizer and inspiratory outlet to protect the inspiratory arm of the ventilator circuit. (**Supplementary Figure 1B**)

### Statistical methods

All coding and data analyses were done using SAS version 9.4 or R version 4.3.2. Continuous variables were summarized as number, mean (standard deviation [SD]), and median (range). The Shapiro-Wilk’s test was used to test for normality within groups, and group comparisons were made using either a Wilcoxon Rank Sum Test or two-sample t-test, as appropriate. Categorical variables were summarized as count and percent, and group comparisons were made using either Pearson’s chi-square test or Fisher’s exact test. Kaplan-Meier and exact log-rank tests were used to compare PICU and hospital survival of those treated and not treated with IP rFVIIa.

## Results

Over the eight-year retrospective period, we identified 31 patients with pulmonary hemorrhage requiring ICU care (**Table 1**). Of the 31 patients, thirteen patients received intrapulmonary IP rFVIIa treatment. Eighteen patients did not receive IP rFVIIa. There were no identified systemic thrombotic events and no obstructed endotracheal tubes requiring exchange in the cohort.

**Table 1:** Entire Cohort (N=31) by PICU Survival.

Variable	Total (n=31)	Survived ICU (n=13)	Died ICU (n=18)	P Value
**Age, years**				0.475
Mean (SD)	9.6 (6.8)	10.7 (6.9)	8.9 (6.9)	.
Median (Range)	10.0 (0.8~23.0)	12.0 (0.8~20.0)	9.5 (0.8~23.0)	.
**Gender**				0.409
Female	17 (54.8%)	6 (46.2%)	11 (61.1%)	.
Male	14 (45.2%)	7 (53.8%)	7 (38.9%)	.
**Diagnosis Pulmonary Hemorrhage**				0.284
BAL	18 (58.1%)	9 (69.2%)	9 (50.0%)	.
Clinical	13 (41.9%)	4 (30.8%)	9 (50.0%)	.
**Primary Dx**				0.288
Brain tumor	1 (3.2%)	1 (7.7%)		.
Non-malignant hematologic disorder	3 (9.7%)	1 (7.7%)	2 (11.1%)	.
Leukemia	21 (67.7%)	7 (53.8%)	14 (77.8%)	.
Lymphoma	1 (3.2%)		1 (5.6%)	.
Primary HLH	1 (3.2%)	1 (7.7%)		.
Solid tumor	4 (12.9%)	3 (23.1%)	1 (5.6%)	.
**Post HCT**				0.111
No	4 (12.9%)	2 (15.4%)	2 (11.1%)	.
Autologous	3 (9.7%)	3 (23.1%)		.
MSD	1 (3.2%)	1 (7.7%)		.
MUD	8 (25.8%)	3 (23.1%)	5 (27.8%)	.
Haploidentical	15 (48.4%)	4 (30.8%)	11 (61.1%)	.
**IP rFVIIa**				0.686
No	18 (58.1%)	7 (53.8%)	11 (61.1%)	.
Yes	13 (41.9%)	6 (46.2%)	7 (38.9%)	.
**Vent-free days**				<.001
Mean (SD)	5.5 (9.2)	13.1 (10.2)	0 (0)	.
Median (Range)	0 (0~25)	17 (0~25)	0 (0~0)	.
**PICU LOS, days**				0.795
Mean (SD)	41.9 (50.3)	48.3 (71.4)	37.2 (28.4)	.
Median (Range)	25 (1~262)	19 (3~262)	41.5 (1~93)	.
**Hospital LOS, days**				0.471
Mean (SD)	65.9 (63.8)	73.1 (90.1)	60.8 (37.3)	.
Median (Range)	46 (3~333)	37 (3~333)	61.5 (4~138)	.
**OSI, pre**				0.733
N	23	10	13	.
Mean (SD)	17.7 (12.1)	15.8 (9.6)	19.1 (13.9)	.
Median (Range)	12.7 (4.5~58.8)	13.7 (4.5~38.8)	12.6 (5.6~58.8)	.
**OI pre**				0.817
N	6	2	4	.
Mean (SD)	32.7 (18.7)	23.4 (29.4)	37.4 (14.5)	.
Median (Range)	30.8 (2.6~59.0)	23.4 (2.6~44.2)	30.8 (29.0~59.0)	.
**PLT count**				0.150
N	29	11	18	.
Mean (SD)	98.3 (55.4)	113.0 (55.1)	89.3 (55.3)	.
Median (Range)	81 (26~230)	103 (41~230)	76 (26~215)	.
**PT**				0.810
Mean (SD)	17.6 (4.0)	17.5 (3.8)	17.8 (4.2)	.
Median (Range)	16.6 (13.0~27.3)	17.1 (13.2~24.9)	16.0 (13.0~27.3)	.
**INR**				0.840
N	29	11	18	.
Mean (SD)	1.6 (0.5)	1.5 (0.5)	1.6 (0.5)	.
Median (Range)	1.4 (1.0~2.8)	1.5 (1.0~2.3)	1.4 (1.1~2.8)	.
**PTT**				0.619
N	28	11	17	.
Mean (SD)	38.1 (11.5)	36.8 (10.4)	39.0 (12.4)	.
Median (Range)	35.6 (22.7~64.7)	35.5 (24.3~57.5)	36.1 (22.7~64.7)	.
**Fibrinogen**				0.997
N	28	10	18	.
Mean (SD)	374.1 (170.3)	374.0 (127.8)	374.2 (193.5)	.
Median (Range)	369 (67~722)	399 (96~531)	338 (67~722)	.

ICU, intensive care unit; HCT, hematopoietic cell transplant; auto, autologous; haplo, haploidentical; MSD, matched sibling donor; MUD, matched unrelated donor; BAL, bronchoalveolar lavage; OSI, oxygenation saturation index; OI, oxygenation index; SD, standard deviation; LOS, length of stay; Vent, ventilator; PLT, platelet; PT, prothrombin time; PTT, partial thromboplastin time; INR, internationalized standard ratio.

Pulmonary hemorrhage was diagnosed by bronchoalveolar lavage in 18 (58.1%) patients and clinically in the remaining patients (defined as hemoptysis, blood visualized from vocal cords on direct laryngoscopy during intubation, bloody secretions from endotracheal tube with diffuse patchy infiltrates on chest radiographic findings and/or hypoxemic respiratory failure).

Overall, 13 of 31 (41.9%) survived to ICU discharge and 11 patients (35.5%) survived to hospital discharge. Severity of illness was evaluated by oxygenation index (OI) or oxygenation saturation index (OSI) when arterial blood gas was not available for OI calculation. The mean OI and OSI in the overall cohort were 32 (SD 18.7) and 17.7 (SD 12.1) respectively. Amongst ICU survivors, the mean OI was 23.4 (SD 29.4) compared to 37.4 (SD 14.5) in non-ICU survivors (p=0.82) and mean OSI was 15.8 (SD 9.6) versus a mean OSI of 19.1 (SD 13.9) in non-survivors (p=0.73). Mean platelet count at time of hemorrhage was 98,000/mm3 (SD 55,400/mm3), with a median of 81,000/mm3 (range 26,000-230,000/mm3) and did not differ significantly between ICU survivors and non-survivors (p=0.15). Mean prothrombin time, partial thromboplastin time, international normalized ratio, and fibrinogen was 17.5 seconds, 38.1 seconds, 1.6 and 374 mg/dL respectively. Ventilator free days was significantly higher in ICU survivors with a mean of 13.1days (SD 10.2) compared to zero days in non-survivors (p=<0.001). PICU length of stay did not differ significantly (**Table 1**).

**Figure 1:**
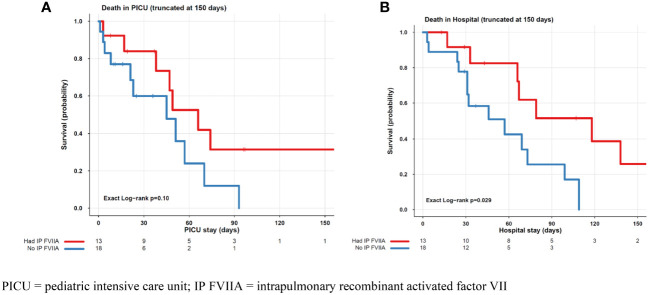
PICU **(A)** and Hospital **(B)** Mortality for Patients Treated and Not Treated with IP FVIIa. PICU, pediatric intensive care unit; IP FVIIA, intrapulmonary recombinant activated factor VII.

Thirteen patients received intrapulmonary rFVIIa, and eighteen patients did not receive IP treatment. Patient characteristics by ICU survival for each treatment group, IP rFVIIa and no IP FVIIa are shown in **Tables 2**, **3**, respectively. Of the 13 patients treated with IP rFVIIa, 6 (46%) survived both ICU and hospital discharge. There was no significant difference in PICU survival between patients treated and not treated with IP-rFVIIa (p=0.1) (**Figure 1A**); however, patients who received IP rFVIIa had lower hospital mortality than patients who were not treated with IP rFVIIa (p=0.029) (**Figure 1B**).

**Table 2:** PICU Survival for Patients Treated with Intrapulmonary FVIIa.

Variable	Total (n=13)	Survived ICU (n=6)	Died ICU (n=7)	P Value
**Age, years**				0.603
N	13	6	7	.
Mean (SD)	9.4 (7.5)	8.1 (7.5)	10.4 (7.9)	.
Median (Range)	9.0 (0.8~23.0)	5.0 (0.8~18.0)	10.0 (1.0~23.0)	.
**Gender**				1.000
Female	5 (38.5%)	2 (33.3%)	3 (42.9%)	.
Male	8 (61.5%)	4 (66.7%)	4 (57.1%)	.
**Primary Diagnosis**				1.000
Non-malignant Hematologic disorder	2 (15.4%)	1 (16.7%)	1 (14.3%)	.
Leukemia	7 (53.8%)	3 (50.0%)	4 (57.1%)	.
Lymphoma	1 (7.7%)		1 (14.3%)	.
Solid tumor	3 (23.1%)	2 (33.3%)	1 (14.3%)	.
**HCT source**				0.125
No	1 (7.7%)	1 (16.7%)		.
Autologous	2 (15.4%)	2 (33.3%)		.
MSD	1 (7.7%)	1 (16.7%)		.
MUD	3 (23.1%)	1 (16.7%)	2 (28.6%)	.
Haploidentical	6 (46.2%)	1 (16.7%)	5 (71.4%)	.
**Diagnosis Pulmonary Hemorrhage**				0.103
BAL	7 (53.8%)	5 (83.3%)	2 (28.6%)	.
Clinical	6 (46.2%)	1 (16.7%)	5 (71.4%)	.
**OSI, pre**				0.219
N	11	5	6	.
Mean (SD)	17.2 (6.7)	14.4 (4.2)	19.6 (7.8)	.
Median (Range)	17.5 (8.2~29.6)	14.7 (8.2~19.0)	20.4 (9.0~29.6)	.
**OI, pre**				NA
N	2	1	1	.
Mean (SD)	44.6 (0.6)	44.2 (.)	45.0 (.)	.
Median (Range)	44.6 (44.2~45.0)	44.2 (44.2~44.2)	45.0 (45.0~45.0)	.
**PICU LOS, days**				0.306
N	13	6	7	.
Mean (SD)	62.5 (67.3)	86.5 (94.0)	42.0 (25.3)	.
Median (Range)	47 (3~262)	66.5 (8~262)	47 (3~74)	.
**Hospital LOS, days**				0.464
N	13	6	7	.
Mean (SD)	92.7 (86.2)	114.5 (120.6)	74 (43)	.
Median (Range)	67 (13~333)	75 (13~333)	67 (17~138)	.
**Vent free days**				0.053
N	13	6	7	.
Mean (SD)	4.7 (9.0)	10.2 (11.4)	0.0 (0.0)	.
Median (Range)	0.0 (0.0~24.0)	8.5 (0.0~24.0)	0.0 (0.0~0.0)	.
**PLT count**				0.561
N	13	6	7	.
Mean (SD)	64.5 (32.3)	70.5 (42.5)	59.4 (22.7)	.
Median (Range)	56 (30~130)	52 (30~130)	74 (31~84)	.
**PT**				0.287
N	13	6	7	.
Mean (SD)	17.7 (4.7)	16.1 (3.4)	19.0 (5.4)	.
Median (Range)	15.4 (13.2~27.3)	15.0 (13.2~22.0)	15.4 (14.1~27.3)	.
**PTT**				0.695
N	13	6	7	.
Mean (SD)	36.1 (12.1)	34.6 (9.0)	37.4 (14.8)	.
Median (Range)	33.4 (22.7~60.0)	34.5 (24.3~50.6)	31.7 (22.7~60.0)	.
**INR**				0.277
N	13	6	7	.
Mean (SD)	1.5 (0.6)	1.4 (0.4)	1.7 (0.6)	.
Median (Range)	1.3 (1.0~2.8)	1.2 (1.0~2.1)	1.3 (1.2~2.8)	.
**Fibrinogen**				0.667
N	13	6	7	.
Mean (SD)	367.0 (136.0)	385.7 (95.9)	351.0 (169.3)	.
Median (Range)	358 (197~694)	395.5 (249~531)	309 (197~694)	.

ICU, intensive care unit; HCT, hematopoietic cell transplant; auto, autologous; haplo, haploidentical; MSD, matched sibling donor; MUD, matched unrelated donor; BAL, bronchoalveolar lavage; OSI, oxygenation saturation index; OI, oxygenation index; SD, standard deviation; LOS, length of stay; Vent, ventilator; PLT, platelet; PT, prothrombin time; PTT, partial thromboplastin time; INR, internationalized standard ratio.

**Table 3:** PICU Survival for Patients Not Treated with Intrapulmonary FVIIa.

Variable	Total (n=18)	Survived ICU (n=7)	Died ICU (n=11)	P Value
**Age (years)**				0.196
N	18	7	11	.
Mean (SD)	10.4 (6.3)	12.9 (6.1)	8.9 (6.1)	.
Median (Range)	11.5 (0.8~20.0)	13.0 (1.0~20.0	10.0 (0.8~19.0	.
**Gender**				0.627
Female	12 (66.7%)	4 (57.1%)	8 (72.7%)	.
Male	6 (33.3%)	3 (42.9%)	3 (27.3%)	.
**Primary Diagnosis**				0.137
Brain tumor	1 (5.6%)	1 (14.3%)		.
Non-malignant Hematologic disorder	1 (5.6%)		1 (9.1%)	.
Leukemia	14 (77.8%)	4 (57.1%)	10 (90.9%)	.
Primary HLH	1 (5.6%)	1 (14.3%)		.
Solid tumor	1 (5.6%)	1 (14.3%)		.
**Post HCT**				0.881
No	3 (16.7%)	1 (14.3%)	2 (18.2%)	.
Autologous	1 (5.6%)	1 (14.3%)		.
MUD	5 (27.8%)	2 (28.6%)	3 (27.3%)	.
Haploidentical	9 (50.0%)	3 (42.9%)	6 (54.5%)	.
**Diagnosis Pulmonary Hemorrhage**				1.000
BAL	11 (61.1%)	4 (57.1%)	7 (63.6%)	.
Clinical	7 (38.9%)	3 (42.9%)	4 (36.4%)	.
**OSI, pre**				1.000
N	12	5	7	.
Mean (SD)	18.2 (15.8)	17.4 (13.4)	18.7 (18.3)	.
Median (Range)	11.4 (4.5~58.8)	11.5 (4.5~38.8)	11.3 (5.6~58.8)	.
**OI, pre**				NA
N	4	1	3	
Mean (SD)	23.3 (13.8)	2.6 (NA)	30.2 (1.6)	
Median (Range)	29.25 (2.6, 32.0)		29.5 (29.0, 32.0)	
**PICU LOS, days**				0.093
N	18	7	11	.
Mean (SD)	26.9 (26.4)	15.6 (11.4)	34.2 (31.0)	.
Median (Range)	18.5 (1.0~93.0)	11.0 (3.0~36.0)	23.0 (1.0~93.0)	.
**Hospital LOS, days**				0.348
N	18	7	11	.
Mean (SD)	46.6 (31.6)	37.6 (30.2)	52.4 (32.5)	.
Median (Range)	34.5 (3.0~109.0)	29.0 (3.0~99.0)	46.0 (4.0~109.0)	.
**Vent free days**				0.004
N	18	7	11	.
Mean (SD)	6.1 (9.6)	15.6 (9.3)	0.0 (0.0)	.
Median (Range)	0.0(0.0~25.0)	19.0(3.0~25.0	0.0(0.0~0.0)	.
**PLT count**				0.770
N	18	7	11	.
Mean (SD)	64.3 (35.6)	68.1 (52.2)	61.8 (22.6)	.
Median (Range)	64.5 (8~130)	81 (8~130)	62 (32~112)	.
**PT**				0.295
N	18	7	11	.
Mean (SD)	17.4 (3.7)	18.6 (4.0)	16.7 (3.4)	.
Median (Range)	16.5 (13.0~24.9)	17.7 (14.3~24.9)	15.5 (13.0~22.5)	.
**PTT**				0.406
N	17	7	10	.
Mean (SD)	53.4 (26.2)	44.7 (17.0)	59.6 (30.5)	.
Median (Range)	41.2 (25.1~101.0)	41.1(27.9~76.0)	46.1 (25.1~101.0)	.
**INR**				0.365
N	18	7	11	.
Mean (SD)	1.5 (0.4)	1.6 (0.5)	1.5 (0.4)	.
Median (Range)	1.4 (1.1~2.3)	1.5 (1.2~2.3)	1.3 (1.1~2.2)	.
**Fibrinogen**				0.934
N	17	6	11	.
Mean (SD)	351.4 (182.3)	346.2 (148.5)	354.2 (205.2)	.
Median (Range)	381 (67~646)	388.5 (96~511)	317 (67~646)	.

ICU, intensive care unit; HCT, hematopoietic cell transplant; auto, autologous; haplo, haploidentical; MSD, matched sibling donor; MUD, matched unrelated donor; BAL, bronchoalveolar lavage; OSI, oxygenation saturation index; OI, oxygenation index; SD, standard deviation; LOS, length of stay; Vent, ventilator; PLT, platelet; PT, prothrombin time; PTT, partial thromboplastin time; INR, internationalized standard ratio.

As we began to implement the use of inhaled TXA clinically after the initiation of study period, a *post hoc* analysis was completed of the patients who received therapy with inhaled TXA. Five patients received inhaled TXA three of whom also received inhaled rFVIIa. All survived to ICU discharge and 80% (n= 4) survived to hospital discharge. There were no adverse events in this group either.

## Discussion

Pulmonary hemorrhage, specifically diffuse alveolar hemorrhage, is a well-recognized pulmonary complication of hematopoietic cell transplantation, occurring in approximately 5% of post-transplant patients (4, 14). It has also been described in the setting of acute myelogenous leukemia (24). Its exact pathogenesis has not been well elucidated but is thought to be from a direct injury to the lung parenchyma followed by a combination of alveolar inflammation and dysregulated cytokine release leading to further damage of the alveolar-capillary membrane (3). The initial lung injury may be secondary to various factors, such as conditioning agents, occult infection, transplant-associated thrombotic microangiopathy, graft-versus-host disease, or idiopathic pneumonia syndrome (3, 15, 25). In patients with acute myelogenous leukemia, this lung injury results from lysis of leukemic cells, which release lysozymes and other enzymes into the circulation (26).

The pathogenesis of DAH is thought to be inflammation-mediated. Therefore, glucocorticoids have historically been the mainstay of therapy, along with other supportive measures such as mechanical ventilation, transfusion of blood products, treatment of potential infections, and extracorporeal membrane oxygenation in rare cases (2–4, 14, 27). While glucocorticoids remain a foundational therapy, optimal dosing is unknown and efficacy is unclear with various publications showing conflicting data. Recently, Chopra et al. showed a survival benefit in the population of pediatric HCT patients who received steroids for management of DAH, while Schoettler et al. found steroids associated with worse survival (23, 28). Steroid use may subject this vulnerable patient population to untoward side-effects such as infection, hypertension, hyperglycemia, and myopathy (4, 14, 29, 30). Therefore, other management strategies are needed.

In recent years, therapies such as rFVIIa and TXA have been used as novel agents in the treatment of post-HCT DAH (17, 18, 23, 31, 32). We report on the use of rFVIIa in the treatment of pulmonary hemorrhage/DAH in the largest cohort of pediatric HCT and oncology patients published to date. We found the use of intrapulmonary rFVIIa both safe. Though not statistically significant, there was a trend towards improved ICU and hospital survival.

Severity of pulmonary illness, defined by OI and OSI at time of pulmonary hemorrhage diagnosis, did not differ significantly between ICU survivors and non-survivors. Propensity for bleeding, as evidenced by mean and median platelet counts at onset of hemorrhage also did not differ between survivors and non-survivors. Although OI may be useful in determining needs for escalating respiratory support, including high frequency oscillatory therapy and/or extracorporeal membrane oxygenation (ECMO), it may not offer insight into chance of survival. The mean and median PLT count at time of diagnosis was 98,000/mm3 and 81,000/mm3, respectively, and did not differ significantly between survivors and non-survivors.

The practice of maintaining PLT count >50,000/mm3 was insufficient to prevent bleeding as 61% (n=19) of pulmonary hemorrhages occurred with a PLT count >50,000/mm3 and furthermore, 39% (n=12) occurred with a PLT count >75,000/mm3. Therefore, normalizing the platelet count is not sufficient to manage pulmonary hemorrhage in this population.

There were no obvious safety issues noted with intrapulmonary administration of the drug. There was no evidence of worsening of oxygenation as the OI remained stable after instillation. There were no episodes of clot formation blocking endotracheal tubes and no patient required reintubation. Systemic levels of rFVIIa were not evaluated as this was not part of our routine practice.

Recombinant activated factor VIIa (rFVIIa) is an intravenous hemostatic agent indicated for the treatment of bleeding episodes and peri-operative management in patients with inherited and acquired hemophilia (33). Hemostasis is achieved by both TF-tissue factor dependent (extrinsic pathway) and independent pathways. The former occurs as rFVIIa binds to TF and activated platelets at sites of tissue injury, thus activating Factor X and Factor IX resulting in thrombin generation and successfully overcoming the TFPI inhibition of activation of factor X. In a TF- independent manner, rFVIIa directly activates Factor X on the surface of activated platelets (34). Recombinant activated factor VII was initially developed for hemophilia A/B patients who also had the presence of an inhibitor (34, 35). Its use is described in a variety of clinical scenarios, such as congenital factor VII deficiency, hepatic dysfunction, post-operative bleeding, and qualitative platelet disorders (36). More recently, nebulized use of rFVIIa has been reported in the treatment of DAH post-HCT. It is thought to promote hemostasis by overcoming an excess of tissue factor pathway inhibitors in inflamed alveoli, thereby restoring thrombin generation (17, 18, 22).

Tranexamic acid (TXA) is a potent anti-fibrinolytic agent, a derivative of the amino acid lysine, that binds to plasminogen, inhibiting its binding to fibrin and thus preventing plasmin activation and subsequent degradation of fibrin clots (37). It has been used as a preventative measure and hemostatic therapy in various clinical conditions including hemophilia, immune thrombocytopenia, trauma, and intraoperatively (38, 39) Its use in pulmonary hemorrhage has been described in a handful of adult and pediatric patients with promising hemostatic results. When administered directly into the airway, it is thought to act by enhancing the activity of remaining anti-fibrinolytic factors at sites of ongoing bleeding while decreasing the risk of adverse effects such as thromboembolic events and neurotoxicity associated with systemically administered TXA (17, 40, 41). Additionally, Schoettler, et al. demonstrated decreased non-relapse mortality with inhaled rFVIIa and inhaled TXA in a retrospective analysis of pediatric HCT patients (23). TXA was delivered by inhalation only, undiluted at either 250 mg (patients < 25 kg) or 500 mg (patients ≥ 25 kg) per dose. All aerosol solutions were delivered using the Aerogen Solo™ vibrating mesh nebulizer, which was placed inline before the humidifier on the inspiratory side of the ventilator circuit. As noted, after encountering problems with ventilator malfunction during inhaled TXA delivery, two Maquet Servo Duo guard filters were placed on the expiratory limb of the ventilator circuit and exchanged immediately upon completion of medication delivery (**Supplementary Figure 1A**). A one-way valve was placed between the nebulizer and inspiratory outlet to protect the inspiratory arm of the ventilator circuit. (**Supplementary Figure 1B**). All filters and equipment were routinely inspected for proper function by the respiratory therapists.

The use of locally instilled, intrapulmonary TXA and rFVIIa for DAH post pediatric HCT was first reported by Bafaqih, et al., in 2015. They reported a series of 18 pediatric patients with post-HCT DAH who were not responsive to conventional therapies and were subsequently treated with IP TXA +/- IP rFVIIa. Of these, 16 patients (89%) achieved hemostasis and 16 patients (89%) survived to ICU discharge. Park, et al., reported a series of 6 pediatric patients with DAH post-HCT treated with intrapulmonary rFVIIa, of which all achieved hemostasis and 4 (67%) were liberated from mechanical ventilation within 7 days.

Although our study demonstrated slightly lower ICU survival rate (58.8%) in comparison to prior studies, our cohort was nearly twice as large as many of the previous reports. Furthermore, both ICU and hospital survival were greater in those who received intrapulmonary procoagulant therapy compared to those who did not. Intrapulmonary therapy with TXA and rFVIIa are components of our comprehensive approach described in **Supplementary Figure 2**. In addition, we institute early bronchoscopy, high mean airway pressure to tamponade alveolar bleeding, glucocorticoid therapy to control inflammation when indicated, and treatment of co-morbidities such as TA-TMA, graft versus host disease, and idiopathic pneumonia syndrome. This two-step hemostasis regimen has also enabled us to support two patients who were refractory to pharmacologic management with rescue cardiopulmonary support (ECMO) (42).

Our study has several important limitations. As a retrospective, single center study our data is limited by chart review and may be incomplete. Additionally, throughout this 8-year review, we lacked a standard treatment approach. As such, clinical practices, and use of TXA and FVIIa were varied, and individual physician decision making factors to offer inhaled treatment or not were not clearly documented to include in the analysis, thereby limiting the interpretation of our results. Furthermore, our population is limited to pediatric oncology and HCT patients, and results may not be generalizable to other populations. Despite these limitations, this study is the largest single center retrospective report of the use of intrapulmonary instillation of rFVIIa in pulmonary hemorrhage seen in oncology and HCT recipients. Our standardized approach is based upon pathophysiological reasoning. There were no safety concerns identified in our patient population and there was a trend towards improved survival. The limited number of subjects likely prevented this trend from being statistically significant as well as the selection of ICU survival as the primary outcome.

This patient population often has co-morbid diagnoses increasing mortality as well as death from underlying cancer and related therapies. Future well designed, multi-center prospective studies evaluating the use of both intrapulmonary TXA and FVIIa are warranted.

We conclude that the use of IP rFVIIa is a safe and feasible therapy in oncology and HCT-associated pulmonary hemorrhage in children. We believe these therapies can be safely used and have the potential to improve survival. They are a welcome addition to the armamentarium of therapies for managing this devastating disease process.

## Conclusion

The use of IP FVIIa is both safe and feasible for the treatment of pulmonary hemorrhage in pediatric oncology and HCT patients. This safety and feasibility were also noted in an exceedingly small cohort of patients treated with inhaled TXA. Intrapulmonary administration of antifibrinolytic therapies should be considered as a treatment option for patients with pulmonary hemorrhage. However, larger prospective studies are warranted to further evaluate the effectiveness and impact on patient outcomes of the aforementioned therapies.

## Data availability statement

The original contributions presented in the study are included in the article/**Supplementary Material**. Further inquiries can be directed to the corresponding author.

## Ethics statement

The studies involving humans were approved by St. Jude Children’s Research Hospital IRB. The studies were conducted in accordance with the local legislation and institutional requirements. Written informed consent for participation was not required from the participants or the participants’ legal guardians/next of kin in accordance with the national legislation and institutional requirements.

## Author contributions

CH: Conceptualization, Data curation, Formal analysis, Funding acquisition, Investigation, Methodology, Project administration, Resources, Software, Supervision, Validation, Visualization, Writing – original draft, Writing – review & editing. JM: Writing – original draft, Writing – review & editing. JG: Formal analysis, Methodology, Writing – review & editing. EH: Writing – review & editing. PB: Investigation, Resources, Writing – review & editing. DH: Writing – review & editing. MH: Writing – review & editing. GK: Formal analysis, Methodology, Writing – review & editing. JR: Investigation, Resources, Writing – review & editing. SS: Investigation, Writing – review & editing. AS: Writing – review & editing. AQ: Investigation, Writing – review & editing. SG: Conceptualization, Data curation, Formal analysis, Funding acquisition, Investigation, Methodology, Project administration, Resources, Software, Supervision, Validation, Visualization, Writing – original draft, Writing – review & editing.
