# Supplementary figures and images for: Intrapulmonary administration of recombinant activated factor VII in pediatric, adolescent, and young adult oncology and hematopoietic cell transplant patients with pulmonary hemorrhage
Source: Front Oncol. 2024 Apr 12;14:1375697. doi: 10.3389/fonc.2024.1375697 (PMC11055461; doi:10.3389/fonc.2024.1375697)

Supplemental Figure 1A

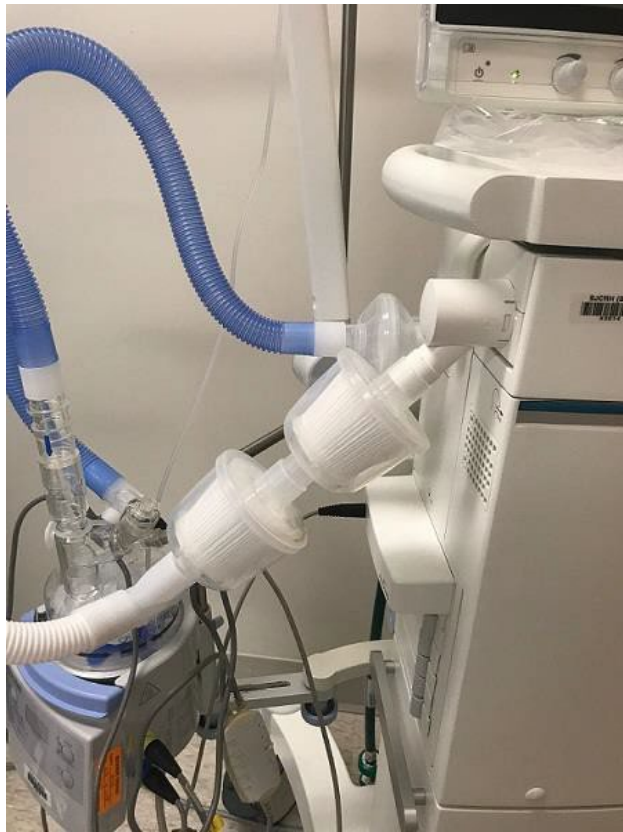

Supplemental Figure 1B

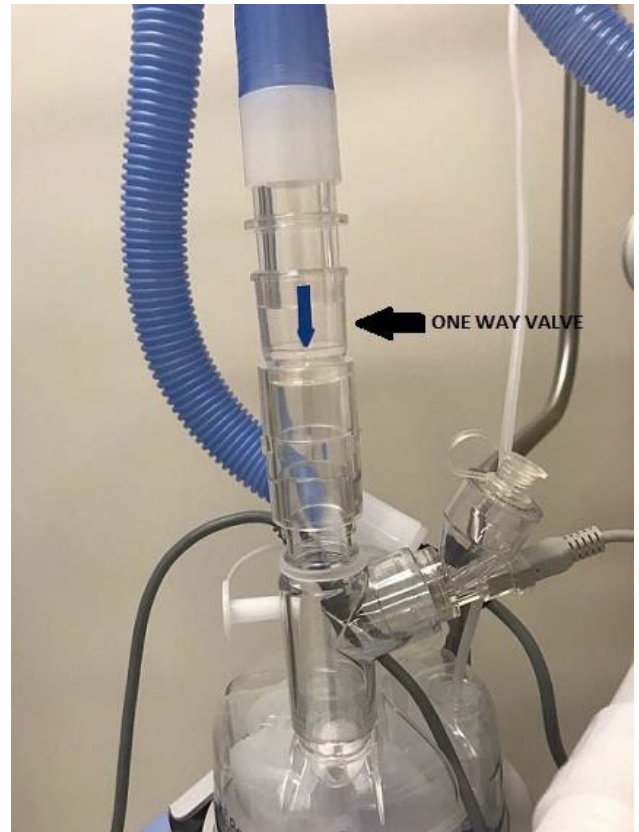

Supplemental Figure 2

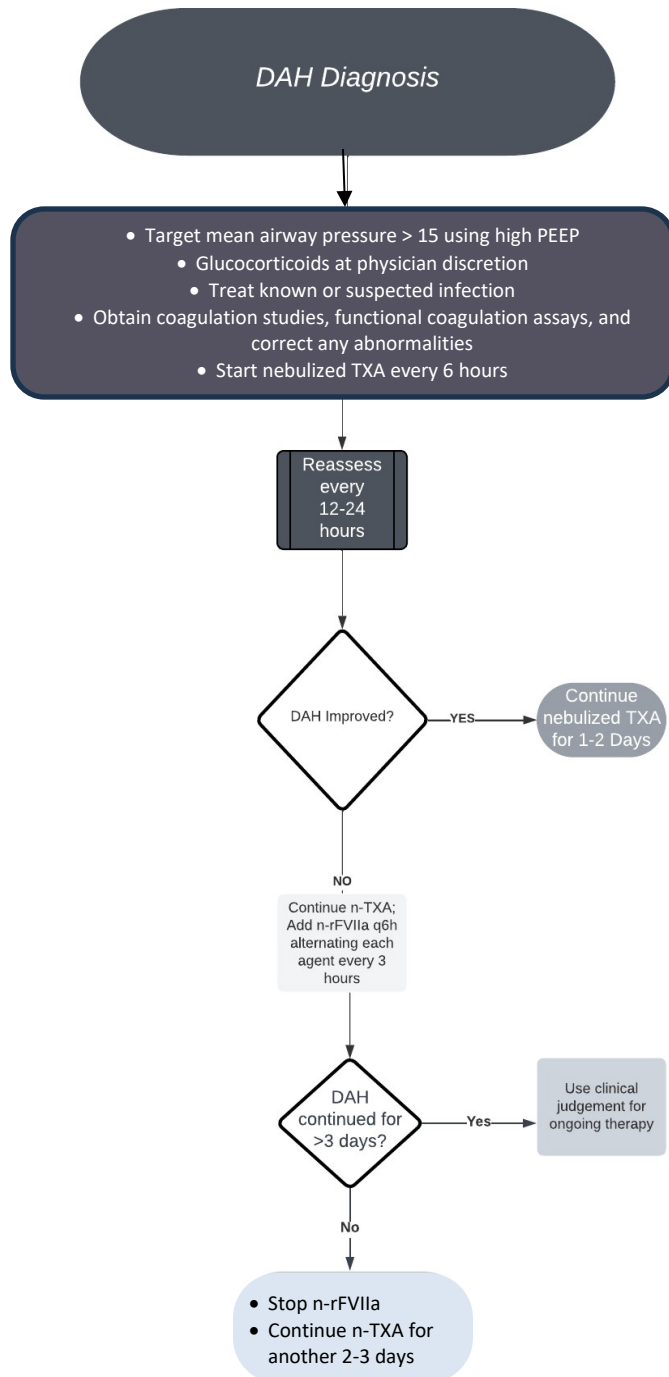

Supplement: Supplementary file 1 [file DataSheet_1.pdf]
